# Factors Associated with the Incidence and Severity of New-Onset Atrial Fibrillation in Adult Critically Ill Patients

**DOI:** 10.1155/2017/8046240

**Published:** 2017-06-15

**Authors:** Péricles A. D. Duarte, Gustavo Elias Leichtweis, Luiza Andriolo, Yasmim A. Delevatti, Amaury C. Jorge, Andreia C. Fumagalli, Luiz Claudio Santos, Cecilia K. Miura, Sergio K. Saito

**Affiliations:** ^1^Hospital do Câncer (UOPECCAN) and Hospital Universitário and Hospital São Lucas, Cascavel, PR, Brazil; ^2^Hospital São Lucas, Cascavel, PR, Brazil; ^3^Curso de Medicina, Faculdade Assis Gurgacz, Cascavel, PR, Brazil; ^4^Hospital Universitário, Cascavel, PR, Brazil; ^5^Hospital Bom Jesus, Toledo, PR, Brazil; ^6^Hospital Nossa Senhora Salete, Cascavel, PR, Brazil; ^7^Hospital Costa Cavalcanti, Foz do Iguaçu, PR, Brazil

## Abstract

**Background:**

Acute Atrial Fibrillation (AF) is common in critically ill patients, with significant morbidity and mortality; however, its incidence and severity in Intensive Care Units (ICUs) from low-income countries are poorly studied. Additionally, impact of vasoactive drugs on its incidence and severity is still not understood. This study aimed to assess epidemiology and risk factors for acute new-onset AF in critically ill adult patients and the role of vasoactive drugs.

**Method:**

Cohort performed in seven general ICUs (including cardiac surgery) in three cities in Paraná State (southern Brazil) for 45 days. Patients were followed until hospital discharge.

**Results:**

Among 430 patients evaluated, the incidence of acute new-onset AF was 11.2%. Patients with AF had higher ICU and hospital mortality. Vasoactive drugs use (norepinephrine and dobutamine) was correlated with higher incidence of AF and higher mortality in patients with AF; vasopressin (though used in few patients) had no effect on development of AF.

**Conclusions:**

In general ICU patients, incidence of new-onset AF was 11.2% with a high impact on morbidity and mortality, particularly associated with the presence of Acute Renal Failure. The use of vasoactive drugs (norepinephrine and dobutamine) could lead to a higher incidence of new-onset AF-associated morbidity and mortality.

## 1. Background

In hospitalized patients, Atrial Fibrillation (AF) is the most common sustained arrhythmia [1, 2], although in most cases this disorder is prior to admission [3]. On the other hand, the pathophysiology, impact, and importance of new-onset AF in the Intensive Care Unit (ICU) is still being investigated more recently [4]. It is known that sepsis and inflammatory process contribute to the development of AF [5], but its multifactorial nature in the critical patient hinders a single explanation for its pathophysiology and consequences. However, it is known that the presence of AF during hospitalization increases morbidity and mortality and hospital costs [2, 6], as well as the incidence of late complications such as heart failure and thromboembolic disorders [7]. Thus, knowledge of incidence and risk factors can lead to strategies that can prevent or reduce the risk of new-onset AF in critical patients. Commonly proposed events which can induce arrhythmia in the ICU include mechanical ventilation, vasoactive drugs, and the systemic inflammation and organ dysfunction [3, 8].

Therefore, the objective of this study was to assess the epidemiological and clinical factors associated with the development of new-onset AF in critically ill patients and the role of vasoactive drugs in this context.

## 2. Material and Methods

This was a cohort study which included all adult patients admitted between November 15 (2015) and January 31 (2016), to seven ICUs of six hospitals (four private, one public, and one philanthropic) in Paraná State, southern Brazil. All the patients admitted during this period were followed daily until being discharged from the hospital, and their data and events were recorded in a specific worksheet.

*Inclusion Criteria*. They were all patients admitted to the ICU, aged 18 years or over, during the stipulated period. 

*Exclusion Criteria*. They were patients who died soon after admission to the ICU (less than 1 hour). 

*Definitions*

- Acute new-onset AF: clinical diagnosis was made by the local assistant staff. Cases with diagnosis or suspicion of chronic AF were excluded. The criterion for diagnosing the arrhythmia was to only identify in the multiparametric monitor or through ECG the AF by the attending physician (the report by the nursing without testimony by the physician was not considered). Surveillance was done by local staff. There was no preestablished arrhythmia maintenance time; therefore, self-limited arrhythmias were considered (as long as identified by the medical team).
- The diagnoses of Acute Respiratory Distress Syndrome (ARDS), sepsis, Acute Renal Failure (ARF), and other ICU diagnoses and comorbidities (e.g., COPD or CHF) were done according to previously established international criteria.

The decisions and strategies regarding the clinical management of patients, including use of respiratory strategies (mechanical ventilation, weaning, and tracheostomy), vasoactive drugs, fluid management, and antibiotic choice, were at the discretion of each local assistant staff. Decisions regarding the management of arrhythmia itself (with eventual attempts to electrical or chemical cardioversion) were also decided by the local assistant team, and the data regarding this management were not included in the study.

*Statistics*. We made descriptive statistics and comparison between percentages with chi-square test. Quantitative variables were compared with Student's* t*-test, Tukey's test, and variance analysis, with *p* < 0.05 being considered significant.

Multivariate analysis by logistic regression was performed to identify two types of variables: (1) factors associated with a higher incidence of AF and (2) (in patients with AF) factors associated with higher mortality. An ROC curve was then constructed for each of the models.

This study was conducted in accordance with the recommendations of Resolution 466/2012 of the Brazilian National Health Council. The project was approved by Faculdade Assis Gurgacz's Permanent Committee on Ethics in Research Involving Human Beings (Cascavel, Brazil).

## 3. Results

A total of 430 patients were admitted in ICUs and included in this study. 59% of the patient population were male with mean age of 58.7 years. The mean Acute Physiology and Chronic Health Evaluation II (APACHE II) score was 17.2. Most of patients (85.1%) had at least one comorbidity (previous disease), with hypertension, obesity, and diabetes mellitus (DM) being the most frequent.  Table 1 shows the clinical and epidemiological profile of the patients.

The incidence of acute new-onset AF was 11.2%. There was a trend to higher admission APACHE II and more surgical elective patients in the AF group. The AF group had more complications, including ARF (with more dialysis needed), ARDS, pneumonia, and vasoactive drugs (VAD), and mechanical ventilation (MV) use. Likewise, the ICU and hospital mortality were higher in the AF group.

Among AF patients, main factors associated with mortality were the presence of complications (mainly ARF and pneumonia) (Table 2), whereas, in the multivariate analysis, the only variable that remained associated with higher mortality was ARF.

**Figure 1:**
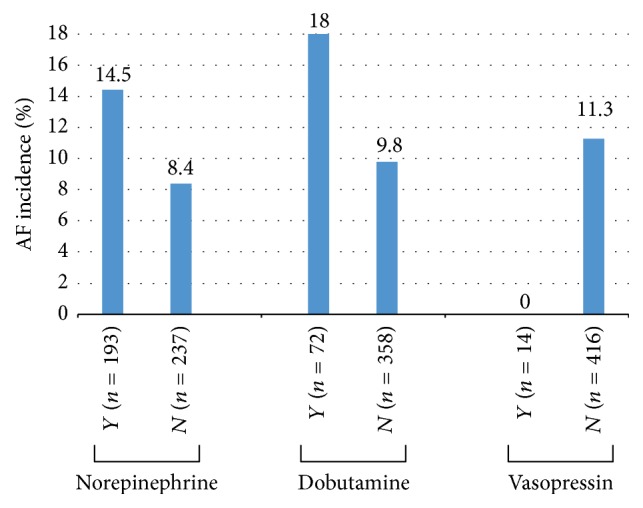
Incidence of AF according to the use of vasoactive drugs (VAD).

**Figure 2:**
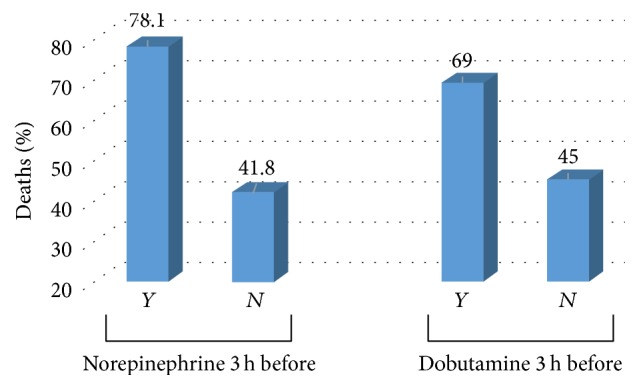
Mortality in patients with AF, according to the use of vasoactive drugs (VAD) immediately before the arrhythmia.

However, the VAD use (mainly dobutamine and norepinephrine) was positively associated with higher incidence of AF and higher mortality in this group. Likewise, the higher the VAD dose, the higher the mortality risk. On the other hand, no link was found between vasopressin use and AF (Figures 1 and 2).

**Figure 3:**
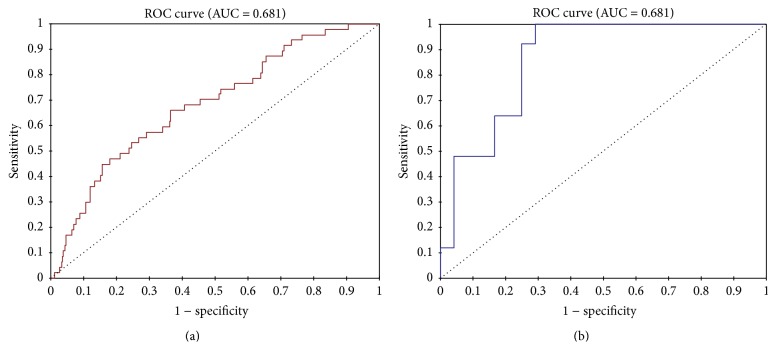
ROC curve of the relationship between Acute Renal Failure (ARF) and incidence of AF (a) and between ARF and mortality in patients with AF (b).

Logistic regression showed that, in the multivariate analysis evaluating the factors involved in the genesis (incidence) and severity (mortality) of acute AF of recent onset, only the presence of ARF was shown as a predictor of incidence and mortality (Table 3 and Figure 3).

## 4. Discussion

In this study (which included 430 surgical and trauma ICU patients) the overall incidence of new-onset AF was 11.2%. Although AF is described in 25% of ICU patients [9], new-onset AF (excluding patients with chronic or previous intermittent AF) in the literature is around 7% [2, 9]. Our study included patients in the postoperative period of cardiac surgery, which could contribute to its higher incidence. However, the AF in non-cardiac surgery patients was also high (10.2%). In patients with trauma, the incidence was 5%, as previously described [10]. The presence of AF also had a higher impact on mortality in both trauma and nontrauma patients (50% and 25.58% in patients with and without AF, resp.).

In the current study, AF was more prevalent in males with age > 65 years as shown previously in both ICU patients and in the general population [2, 4, 11]. This was independent of etiology: in trauma patients, there was no incidence of AF in females. AF was also more frequent in severe patients. Although there were no statistically significant differences, the admission APACHE II was higher in patients with AF. Excluding cardiac surgery patients, APACHE score could predict AF (*p* = 0.044).

We could not find any correlation of comorbidity, smoking, or alcoholism with AF. The literature is controversial as to the relationship between comorbidities and the incidence of AF in critical patients with some studies showing positive correlation between cardiovascular diseases and AF in the ICU [12] with other studies (including the present study) finding no correlation with comorbidities, especially in patients with sepsis [2, 9, 13].

The presence of AF had a strong correlation with the outcomes of patients in the ICU: MV length of time was higher, and the mortality (both in the ICU and in hospital) was higher in patients who had acute AF, as reported [2, 14].

In the multivariate analysis, only the presence of ARF remained correlated with the incidence and severity (mortality) of AF.

It is often difficult to determine if the presence of AF is a consequence of severity in some patients, reflecting a greater systemic inflammatory process, greater number of organic dysfunctions, and cardiovascular overload. This may be suggested in the present study by the strong association between some complications (such as ARF, ARDS, and nosocomial pneumonia) and the incidence of AF in our study. It is well recognized that the presence and amount of organ dysfunction are strongly associated with the incidence of AF in both cardiac surgery [15] and septic [2] or general ICU patients [12]. Therefore, it is a universal complication common to ICU patients: either due to association with obvious cardiac conditions (such as in elderly, cardiopathies, and postoperative cardiac surgery patients) or in patients without previous factors but with high severity, intense inflammatory process, and multiple organ dysfunctions such as sepsis, postoperative, and trauma. The same difficulty of evaluation (causal association or marker of severity) could be said of the use of some procedures, such as pulmonary artery catheter. However, in the current study, no patient underwent this procedure.

Another possible mechanism to explain the effects of AF, which we explore in this study, is the use (and possible excess) of vasopressor drugs, particularly adrenergic drugs. In this study, an important factor in the incidence of AF was the use of vasoactive drugs, specifically the two most used in these hospitals during the period—norepinephrine and dobutamine (Figure 1). These drugs at higher dosages and longer use were associated with the incidence of AF, and their higher dosages and longer use were associated with increased mortality in AF group. Also, apart from being used as therapeutic strategy for hemodynamic instability after AF, their use before the incidence of AF was high and correlated with mortality in current study. It could, therefore, have implications in the genesis of the arrhythmia. The possible role of adrenergic drugs (dobutamine, dopamine, and norepinephrine) in the development of acute AF has been observed in situations as varied as stress-echocardiography [16], postoperative cardiac surgery [17], and trauma [18]. It is speculated that one of the mechanisms of genesis of AF could be an excess of catecholamines or an increased sensitivity to them [19]. Vasopressin could also trigger AF [20], although it may have a lower ability to induce arrhythmias in critical patients [21, 22]. In our study, no patient in vasopressin use developed AF (Table 2 and Figure 1). It should be noted, however, that the present study was not designed to detect a possible effect of vasopressin or other vasoactive drugs on arrhythmia incidence, severity, or mortality. Therefore, this study has no statistical power to categorically state that the use of any specific type of vasopressor directly influences the genesis of acute AF—particularly since few patients used vasopressin. However, the data from this study may contribute to better understanding and management of the use of VAD and prevention of AF, since our results are similar to those of other studies [4, 11, 12].

However, when evaluating a critical patient, it is difficult to assess by cohort studies whether the use of vasoactive drugs induced AF or its use was concomitant or even secondary to arrhythmia (due to circulatory instability). In our study, we found that the use of catecholamines (norepinephrine and dobutamine) immediately prior to the AF was not only related to its incidence but also correlated to its severity (with respect to the hospital mortality). In addition, higher dose of these drugs was also correlated with mortality. No patient with vasopressin use (although its use was limited) developed AF. Therefore, this study detected a possible temporal association between the use of vasoactive adrenergic drugs (and their dosage) with the appearance and severity (mortality) of AF in critically ill patients and a possible protective role of vasopressin.

This study has some limitations, some of them inherent to its nature. Because it was an observational study, the impact of arrhythmia management and prevention strategies could not be evaluated. Also, some variables (such as the presence of obstructive sleep apnea or the type of trauma [thorax × nonthorax]) were not discriminated, due to the design of the study. In addition, a reasonable heterogeneity could arise in evaluation and management of patients, as it is a multicenter study. The diagnostic criterion for AF itself was based on clinical detection, which is known to have lower sensitivity than specific screening with 24-h Holter [11]. We also did not evaluate the correlation between the myocardial function/ejection fraction and the incidence or severity of the arrhythmia, since only a few centers performed an echocardiogram in these patients. However, the objective of the study was precisely a real-life assessment of different ICUs (teaching and nonteaching) and different types of patients. Anyway, this study highlights the frequency of this arrhythmia in general ICUs, and the authors emphasize the importance for eventual supplementary functional cardiac assessment in this subgroup of patients.

## 5. Conclusions

In a heterogeneous group of adult ICU patients, incidence of new-onset AF was 11.2% with a high impact on morbidity and mortality, particularly associated with the presence of Acute Renal Failure. We found that the use of vasoactive (adrenergic) drugs, especially norepinephrine and dobutamine, was correlated to higher incidence and severity of acute AF, whereas vasopressin (although used in few patients) had no effect on development of AF.

## Figures and Tables

**Table 1:** Demographic, clinical, and outcome data.

	Total	AF (+)	AF (−)	*p*
	*n* = 430	*n* = 48	*n* = 382	
Male gender, *n* (%)	254 (59.1%)	34 (70.8%)	220 (57.6%)	0.109
Age, years, mean ± SD	58.7 ± 18.00	63.0 ± 17.40	58.2 ± 18.26	0.086
≤40	72 (16.8%)	8 (16.6%)	64 (16,7%)	0.023
41–65	191 (44.4%)	15 (31.3%)	176 (46.1%)	
>65	167 (38.8%)	25 (52.1%)	142 (34.2%)	
APACHE II, mean ± SD	17.2 ± 8.36	19.2 ± 9.27	17.0 ± 8.21	0.090
≤5	12 (2.8%)	2 (4.2%)	10 (2.6%)	0.063
6–10	93 (21.6%)	4 (8.2%)	89 (23.3%)	
11–20	189 (43.9%)	27 (56.3%)	162 (42.4%)	
21–30	103 (23.9%)	8 (16.7%)	95 (24.9%)	
>30	33 (7.8%)	7 (14.6%)	26 (6.8%)	
Admission etiology, *n* (%)	—	—	—	—
Trauma	35 (8.1%)	2 (4.2%)	33 (8.6%)	0.056
Medical	196 (45.6%)	24 (50%)	172 (45.0%)	
Surgical, elective	150 (34.9%)	21 (43.7%)	129 (33.8%)	
Surgical, urgency, nontrauma	49 (11.4%)	1 (2.1%)	48 (12.6%)	
Comorbidities, *n* (%)	—	—	—	—
None	64 (14.9%)	5 (10.4%)	59 (15.4%)	0.479
Hypertension	201 (46.7%)	27 (56.2%)	174 (45.5%)	0.212
DM	70 (16.3%)	8 (16.7%)	62 (16.2%)	0.895
Cancer, actual	69 (16.0%)	6 (12.5%)	63 (16.5%)	0.616
Obesity (BMI ≥ 30)	51 (11.9%)	4 (8.3%)	47 (12.3%)	0.572
CHF functional class III or IV	43 (10%)	7 (14.6%)	36 (9.4%)	0.385
COPD	34 (7.9%)	6 (12.5%)	28 (7.3%)	0.332
CRF	30 (7.0%)	4 (8.3%)	26 (6.8%)	0.927
CRF with dialysis	6 (1.4%)	2 (4.2%)	4 (1.0%)	0.277
Severe neurological sequelae	5 (1.2%)	1 (2.1%)	4 (1.0%)	0.935
Smoking, actual or recent, *n* (%)	35 (8.1%)	6 (12.5%)	29 (7.6%)	0.372
Alcohol use, actual or recent, *n* (%)	16 (3.7%)	1 (2.1%)	15 (3.9%)	0.818
Complications, *n* (%)	—	—	—	—
ARF	145 (33.7%)	26 (54.2%)	119 (31.2%)	0.003
Dialysis	38 (8.8%)	9 (18.8%)	29 (7.6%)	0.022
ARDS	77 (17.9%)	14 (29.2%)	63 (16.5%)	0.050
Pneumonia	74 (17.2%)	15 (31.3%)	59 (15.4%)	0.011
Invasive MV	228 (53.0%)	27 (56.2%)	201 (52.6%)	0.747
Tracheostomy	52 (12.1%)	9 (18.8%)	43 (11.2%)	0.205
VAD use (any), *n* (%)	228 (53.0%)	31 (64.6%)	197 (51.6%)	0.121
Dobutamine	72 (16.7%)	13 (27.1%)	59 (15.4%)	0.067
Dopamine	4 (0.9%)	0	4 (1.0%)	0.928
Vasopressin	14 (3.3%)	0	14 (3.7%)	0.360
Norepinephrine	193 (44.9%)	28 (58.3%)	165 (43.2%)	0.067
Total time Nor, days, mean ± SD	1.78 ± 3.23	2.81 ± 3.84	1.65 ± 3.13	0.019
0–2	316 (73.5%)	27 (56.2%)	289 (75.6%)	0,007
>2	114 (26.5%)	21 (43.8%)	93 (24.4%)	
Maximal dose Nor (*μ*g/kg/min), mean ± SD	0.43 ± 3.92	1.93 ± 11.51	0.24 ± 0.50	0.004
0–0,20	322 (74.9%)	28 (58.3%)	294 (77.0%)	0.008
>0,20	108 (25.1%)	20 (41.7%)	88 (23.0%)	
Invasive MV, days, mean ± SD	2.64 ± 5.33	4.43 ± 7.17	2.42 ± 5.02	0.014
0 (no use)	202 (47.0%)	21 (43.8%)	181 (47.4%)	0.028
1	81 (18.8%)	5 (10.4%)	76 (19.9%)	
2–5	83 (19.3%)	10 (20.8%)	73 (19.1%)	
6–10	37 (8.6%)	5 (10.4%)	32 (8.4%)	
>10	27 (6.3%)	7 (14.6%)	20 (5.2%)	
ICU mortality, *n* (%)	25.6%	50%	22.5%	<0.001
Hospital mortality, *n* (%)	30.2%	52.08%	27.5%	<0.001

AF: Atrial Fibrillation. SD: standard deviation. APACHE: Acute Physiology and Chronic Health Evaluation. BMI: Body Mass Index. COPD: Chronic Obstructive Pulmonary Disease. DM: diabetes mellitus. CHF: Chronic Heart Failure. CRF: Chronic Renal Failure. ARF: Acute Renal Failure. ARDS: Acute Respiratory Distress Syndrome. MV: mechanical ventilation. VAD: vasoactive drugs. Nor: Norepinephrine. ICU: Intensive Care Unit.

**Table 2:** Patients with AF: risk factors for hospital mortality.

	Total with AF	Alive, hospital	Dead, hospital	*p*
	*n* = 48	*n* = 23	*n* = 25	
Male gender, *n* (%)	34 (70.83%)	18 (78.26%)	16 (64.0%)	0.442
Age, years, mean ± SD	63.0 ± 17.40	63.39 ± 14.92	62.64 ± 19.72	0.883
≤40	8 (16.55%)	4 (17.39%)	4 (16.0%)	0.796
41–65	15 (31.25%)	7 (30.43%)	8 (32.0%)	
>65	25 (52.08%)	12 (52.17%)	13 (52.0%)	
APACHE, mean ± SD	19.18 ± 9.27	18.69 ± 8.72	19.64 ± 9.91	0.727
Admission etiology, *n* (%)	—	—	—	—
Trauma	2 (4.16%)	0 (0%)	2 (8%)	0.156
Medical	24 (50%)	10 (43.47%)	14 (56%)	
Surgical, elective	21 (43.75%)	13 (56.52%)	8 (32%)	
Surgical, urgency, nontrauma	1 (2.09%)	0 (0%)	1 (4%)	
Comorbidities, *n* (%)	—	—	—	—
None	5 (10.41%)	1 (4.34%)	4 (16%)	0.396
Obesity (BMI ≥ 30)	4 (8.33%)	1 (4.34%)	3 (12%)	0.662
COPD	6 (12.5%)	3 (13.04%)	3 (12%)	0.743
DM	8 (16.66%)	4 (17.39%)	4 (16%)	0.796
CHF, NYHA class III or IV	7 (14.58%)	3 (13.04%)	4 (16%)	0.905
CRF with dialysis	4 (8.33%)	1 (4.34%)	3 (12%)	0.662
Cancer, actual	6 (12.5%)	2 (8.69%)	4 (16%)	0.743
Severe neurological sequelae	1 (2.08%)	1 (4.34%)	0 (0%)	0.968
CRF	2 (4.16%)	1 (4.34%)	1 (4%)	0.506
Smoking, actual or recent	6 (12.5%)	4 (17.39%)	2 (8%)	0.585
Alcohol use, actual or recent	1 (2.08%)	1 (4.34%)	0 (0%)	0.968
Complications, *n* (%)	—	—	—	—
ARF	26 (54.16%)	6 (26.09%)	20 (80%)	<0.001
Dialysis	9 (18.75%)	3 (13.04%)	6 (24%)	0.547
ARDS	14 (29.16%)	4 (17.39%)	10 (40%)	0.160
Pneumonia	15 (31.25%)	6 (26.08%)	9 (36%)	0.668
Invasive MV	27 (56.25%)	8 (34.78%)	19 (76%)	0.010
Tracheostomy	9 (18.75%)	3 (13.04%)	6 (24%)	0.547
Norepinephrine use	28 (58.33%)	11 (47.82%)	17 (68%)	0.261
Dobutamine use	13 (27.08%)	4 (17.39%)	9 (36%)	0.261
Total time Nor, days, mean ± SD	2.81 ± 3.84	1.65 ± 2.01	3.88 ± 4.76	0.043
0–7	43 (89.6%)	23 (100%)	20 (80.0%)	0.073
>7	05 (10.4%)	0	05 (20.0%)	
Maximal dose Nor (*μ*g/kg/min), mean ± SD	0.29 ± 0.42	0.19 ± 0.21	0.43 ± 0.51	0.041
0–0,50	37 (77.1%)	21 (91.3%)	16 (64.0%)	0.057
>0,50	11 (22.9%)	02 (8.7%)	09 (36.0%)	
Using Nor 3 h before AF, *n* (%)	14 (29.2%)	03 (13.0%)	11 (44,0%)	0.041
Dose Nor 3 h before AF (*μ*g/kg/min), mean ± SD	0.12 ± 0.26	0.04 ± 0.11	0,19 ± 0,33	0.044
Zero (not using)	34 (70.83%)	20 (86.95%)	14 (56%)	0.041
0,01–0,05	0 (0%)	0 (0%)	0 (0%)	
0,06–0,10	2 (4.16%)	0 (0%)	2 (8%)	
0,11–0,20	5 (10.41%)	1 (4.34%)	4 (16%)	
0,21–0,50	3 (6.25%)	2 (8.69%)	1 (4%)	
0,51–0,80	2 (4.16%)	0 (0%)	2 (8%)	
0,81–1,0	1 (1.08%)	0 (0%)	1 (4%)	
>1,0	1 (1.08%)	0 (0%)	1 (4%)	
Using Dobuta before AF, *n* (%)	13 (27.1%)	04 (17.4%)	09 (36.0%)	0.261
Total time Dobuta, days, mean ± SD	1.14 ± 2.27	0.69 ± 1.79	1.56 ± 2.61	0.188
Maximal dose Dobuta (*μ*g/kg/min), mean ± SD	2.39 ± 4.56	1.67 ± 3.91	3.06 ± 5.08	0.297
Dose Dobuta 3 h before AF (*μ*g/kg/min), mean ± SD	1.42 ± 3.01	0.85 ± 2.45	1.91 ± 3.43	0.228
Invasive MV, days, mean ± SD	4.43 ± 7.17	3.43 ± 8.06	5.36 ± 6.27	0.357
Time (days) between ICU admission and AF, mean ± SD	9.35 ± 8.67	8.47 ± 7.92	10.16 ± 9.41	0.506
Zero (Admission day)	8 (16.7%)	5 (21.7%)	3 (12%)	0.379
1	7 (14.6%)	2 (8.7%)	5 (20%)	
2–5	5 (10.4%)	2 (8.7%)	3 (12%)	
6–10	9 (18.8%)	6 (26.1%)	3 (12%)	
>11	19 (39.6%)	8 (34.8%)	11 (44%)	
ICU LOT, days, mean ± SD	7.60 ± 9.35	7.26 ± 11.21	7.92 ± 7.47	0.810
Hospital LOT, days, mean ± SD	12,15 ± 15,38	12.30 ± 16.09	11.96 ± 15.02	0.940

AF: Atrial Fibrillation. SD: standard deviation. APACHE: Acute Physiology and Chronic Health Evaluation. BMI: Body Mass Index. COPD: Chronic Obstructive Pulmonary Disease. DM: Diabetes Mellitus. CHF: Chronic Heart Failure. CRF: Chronic Renal Failure. ARF: Acute Renal Failure. ARDS: Acute Respiratory Distress Syndrome. MV: mechanical ventilation. VAD: vasoactive drugs. Nor: Norepinephrine. ICU: Intensive Care Unit.

**Table 3:** Parameters obtained for the model created through the application of logistic regression in order to find the factors that predispose to Atrial Fibrillation.

Source	Value	Standard error	Wald X^2^	Pr > X^2^	Odds ratio	CI odds (95%)
ARF	1.04	0.31	10.98	<0.0001	2.84	1.53–5.26
