# Corrigendum to “Factors Associated with the Incidence and Severity of New-Onset Atrial Fibrillation in Adult Critically Ill Patients”

**DOI:** 10.1155/2019/5710734

**Published:** 2019-01-29

**Authors:** Péricles A. D. Duarte, Gustavo Elias Leichtweis, Luiza Andriolo, Yasmim A. Delevatti, Amaury C. Jorge, Andreia C. Fumagalli, Luiz Claudio Santos, Cecilia K. Miura, Sergio K. Saito

**Affiliations:** ^1^Hospital do Câncer (UOPECCAN) and Hospital Universitário and Hospital São Lucas, Cascavel, PR, Brazil; ^2^Hospital São Lucas, Cascavel, PR, Brazil; ^3^Curso de Medicina, Faculdade Assis Gurgacz, Cascavel, PR, Brazil; ^4^Hospital Universitário, Cascavel, PR, Brazil; ^5^Hospital Bom Jesus, Toledo, PR, Brazil; ^6^Hospital Nossa Senhora Salete, Cascavel, PR, Brazil; ^7^Hospital Costa Cavalcanti, Foz do Iguaçu, PR, Brazil

In the article titled “Factors Associated with the Incidence and Severity of New-Onset Atrial Fibrillation in Adult Critically Ill Patients” [1], the sentence “However, the AF in non-cardiac surgery patients was also high (10.2%)” in the Discussion should be rephrased to “However, the incidence of AF in all patients (medical or surgical) after excluding those who were not submitted to cardiac surgery was also high (10.2%)” to avoid any misinterpretation.
